# Supplementary material for: Lysyl Oxidase-Like 2 Protects against Progressive and Aging Related Knee Joint Osteoarthritis in Mice
Source: Int J Mol Sci. 2019 Sep 27;20(19):4798. doi: 10.3390/ijms20194798 (PMC6801581; doi:10.3390/ijms20194798)
Supplement: Supplementary file 1 [file ijms-20-04798-s001.pdf]

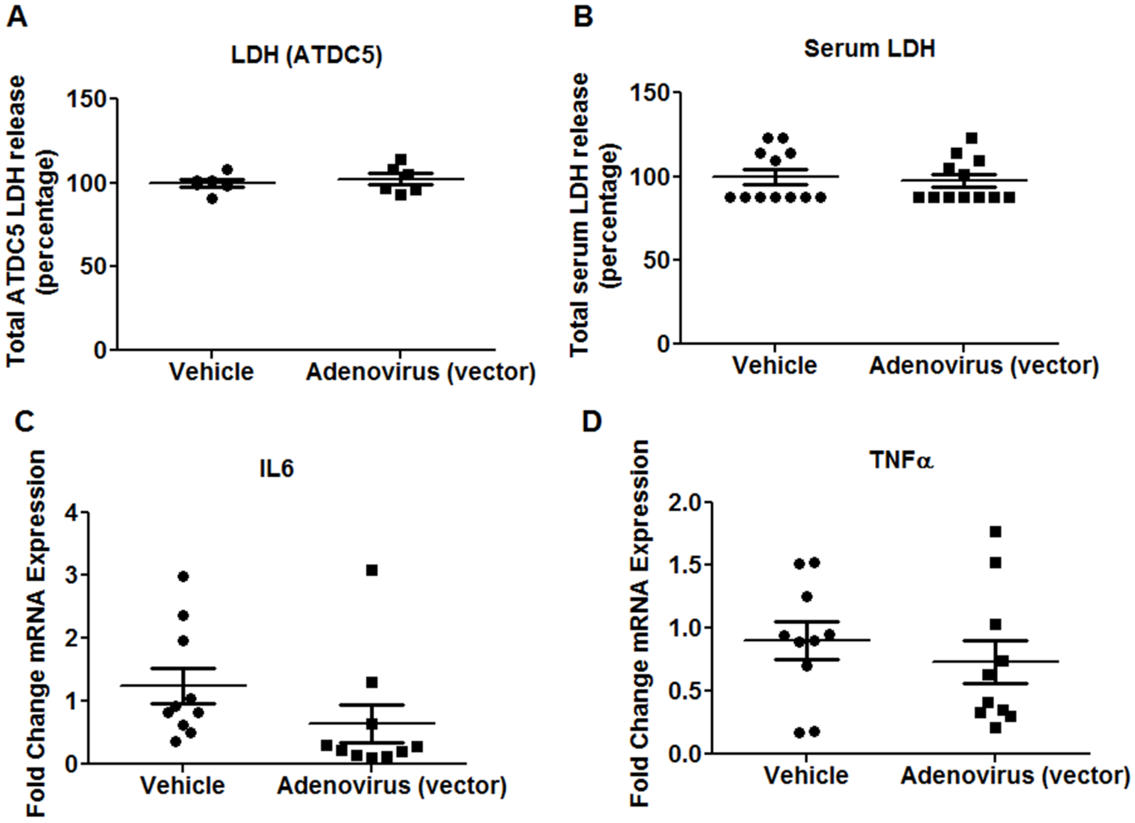

Figure S1. Adenovirus vector injection does not induce adverse effects. A) Cytotoxicity in ATDC5 cells evaluted by LDH assay. B) Total LDH activity in serum samples injected with vehicle or adenvorus vector (adv-RFP-LOXL2) evaluted by LDH assay. C) IL6 mRNA expression in knee joint of mice injected with vehicle or adenvorus vector (adv-RFP-LOXL2). D) TNF*α* mRNA expression in knee joint of mice injected with vehicle or adenvorus vector (adv-RFP-LOXL2). .
